# Streamlined calculation of kidney function using dynamic contrast-enhanced MRI with population-based arterial input function and a whole-kidney model

**DOI:** 10.1186/s41747-026-00704-3

**Published:** 2026-04-21

**Authors:** Xin Mu, Mira M. Liu, Haitham Al-Mubarak, Paul Kennedy, Philip Robson, Jordan Cuevas, Bernd Kuhn, Ketan Badani, Bachir Taouli, Sara Lewis, Octavia Bane

**Affiliations:** 1 BioMedical Engineering and Imaging Institute, Icahn School of Medicine at Mount Sinai, New York, NY USA; 2 Department of Diagnostic, Molecular and Interventional Radiology, Icahn School of Medicine at Mount Sinai, Mount Sinai Hospital, New York, NY USA; 3 Siemens Healthcare, Erlangen, Germany; 4 Department of Urology, Icahn School of Medicine at Mount Sinai, New York, NY USA

**Keywords:** Kidney, Pharmacokinetic model, Glomerular filtration rate, Magnetic resonance imaging, Renal plasma flow

## Abstract

**Objectives:**

Dynamic contrast-enhanced (DCE) magnetic resonance imaging (MRI) can assess kidney function, but artifacts and complex post-processing limit its use. We calculated estimated glomerular filtration rate (eGFR) and renal plasma flow (RPF) by combining a population-based arterial input function (pAIF) with a whole-kidney pharmacokinetic model (WKPM). We also compared DCE-MRI eGFR and RPF with serum eGFR and arterial spin labeling (ASL) derived RPF, respectively.

**Materials and methods:**

In a prospective single-center study, 43 patients (30 M/13 F, 59.0 ± 11.8 y) with renal masses underwent multiparametric 1.5-T MRI, before and 3 months after nephrectomy (*n* = 15), including coronal, fat-saturated volumetric DCE-MRI (5-s temporal resolution) and background-suppressed pseudocontinuous ASL. DCE-MRI eGFR and RPF were measured by WKPM, incorporating individual-based arterial input function (iAIF) and population-based arterial input function (pAIF) as inputs. Pearson correlation, Bland-Altman analysis, and Mann-Whitney *U* statistics were used.

**Results:**

Serum eGFR (mean 67.55 mL/min/1.73 m²) and DCE-MRI (mean eGFR pAIF 59.49, iAIF 63.60 mL/min/1.73 m²) were measured in 51 MRIs: correlation with serum eGFR was stronger for pAIF (*r* = 0.61, *p* < 0.001) than iAIF (*r* = 0.33, *p* = 0.018), with comparable Bland-Altman bias (-11.9% and -9.1%, respectively). RPF was measured by both DCE-MRI and ASL in 21 MRIs: mean RPF was 229.3 (ASL), 229.7 (pAIF), and 390.4 (iAIF) mL/min (*p* = 0.018). Correlation of pAIF RPF with ASL-derived RPF (*r* = 0.65, *p* < 0.001) was stronger than for iAIF RPF (r = 0.53, *p* = 0.014), with lower Bland-Altman bias (pAIF -1.0% *versus* iAIF 39.5%).

**Conclusion:**

DCE-MRI using pAIF and WKPM provides simplified, robust single-kidney function estimates.

**Relevance statement:**

This study proposes a simplified DCE-MRI post-processing method using a population-based arterial input function combined with a whole-kidney pharmacokinetic model. It avoids complex corticomedullary segmentation and minimizes aortic region-of-interest variability, and enables clinically feasible estimation of single-kidney function, supporting broader adoption of renal DCE-MRI in clinical practice.

**Key Points:**

- Population-based arterial input function reduces inter-observer variability and sensitivity to aortic region-of-interest placement artifacts.
- Whole-kidney modeling avoids complex segmentation of the cortex and medulla regions.
- DCE-MRI using population-AIF and whole-kidney modeling yields eGFR and RPF significantly correlated with serum and ASL references.
- Streamlined post-processing workflow supports broader routine clinical use of DCE-MRI.

**Graphical Abstract:**

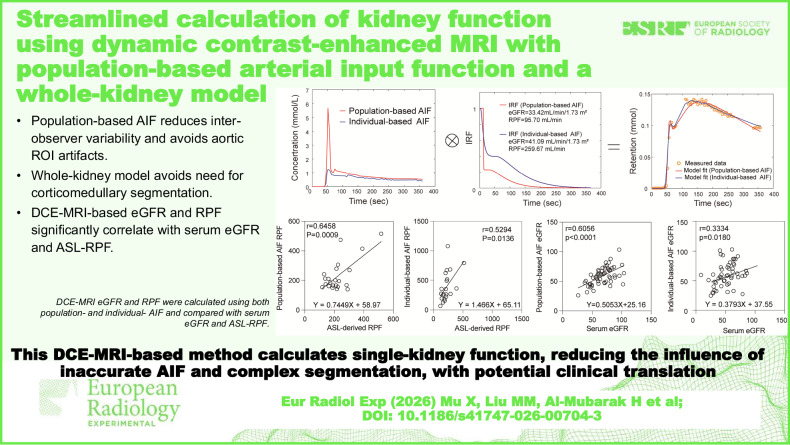

## Background

Glomerular filtration rate (GFR) and renal plasma flow (RPF) are two essential parameters for measuring renal function [1]. Reference standard methods for measurement of these physiological parameters, radionuclide clearance for GFR and para-aminohippuric acid clearance for RPF, are complex, costly, require specialized equipment, and are inconvenient for patients [2, 3]. Serum eGFR calculation is the most widely used clinical measure of renal function; however, it does not measure single-kidney function, which is informative for planning surgical management of renal masses, for the assessment of renal transplant donors, or in patients with unilateral kidney disease [4]. MRI is well-suited for repeat evaluation and detailed anatomical and functional assessments of the kidneys, due to the noninvasive, radiation-free technique and excellent soft tissue contrast resolution [5]. A well-validated imaging method for assessing perfusion, arterial spin labeling (ASL) does not require gadolinium-based contrast agents, and is consequently safe for patients with renal impairment, but it has intrinsically low signal-to-noise-ratio and is easily influenced by respiratory motion [6]. Dynamic contrast-enhanced (DCE)-MRI can be used to calculate both single kidney eGFR and RPF, but is not used widely in clinical practice, due to complex processing [3, 7–9].

DCE-MRI processing involves two labor-intensive and time-consuming tasks: the tracer kinetic modeling and derivation of the concentration curves of the aorta, known as the arterial input function (AIF) and of target tissues, hindering clinical adoption [10]. Common renal tracer kinetic models used to measure eGFR and RPF, such as the one-compartment, two-compartment and three-compartment models, represent the simplified versions of the complete multicompartmental model of the kidney, none of which are widely validated or implemented clinically [11–14]. The one-compartment (Baumann-Rudin) model does not measure eGFR directly, but rather provides an index proportional to GFR [15]. Two-compartment models mainly include the Patlak–Rutland model, the various modified two-compartment models, as well as the whole-kidney model with a more elaborate parallel two-pathway [15, 16]. The Patlak model, though attractive for its simplicity, is highly sensitive to the arbitrary selection of the time window used for slope calculation [16], while the modified two-compartment models either overestimated the eGFR or fitted the data poorly [15–17]. Although more advanced three-compartment renal models offer a closer representation of kidney physiology and theoretically enhance the accuracy of those parameters, they also require separate segmentation of the cortex and medulla, and consequently, more precise image registration and segmentation [12–14]. Especially in diseased kidneys, the indistinct boundaries among the cortex, medulla, renal pelvis, and tumors, compounded by imaging artifacts, further complicate segmentation and increase the post-processing workload [11, 18–20]. Under such conditions, automatic registration and segmentation may fail to maintain acceptable accuracy, making manual correction by the radiologist unavoidable. The whole-kidney model treats the kidney parenchyma as a single unit and combines the signal intensities of the cortex and medulla, thus simplifying the segmentation of the whole kidney [11]. Eliminating cortico-medullary segmentation challenges, the simple whole-kidney model improves the DCE-MRI post-processing pipeline and enhances potential clinical adoption of DCE-MRI [1, 11]. Overall, the whole-kidney model provides more physiologically realistic behavior than one-compartment models, requiring only a simpler whole-kidney region of interest (ROI) compared with three-compartment models.

AIF also plays a crucial role in DCE-MRI processing, yet its accurate acquisition remains technically challenging [10]. Individual-based AIF (iAIF), also referred to as image-derived, subject-specific AIF, is frequently used in tissue with a large arterial vessel within the depicted field of view [10]. However, the accuracy of iAIF can be influenced by pulse sequence parameters (such as temporal resolution, uniformity of the B_1_ field), MRI artifacts (such as partial volume effects, inflow effects, blood flow turbulence, etc.) and the dose of contrast agent [10, 21, 22]. Also, the ROI selection in the aorta lacks standardization, yet the size and location of the ROI may influence the model-based perfusion quantification [23, 24]. Given these challenges, previous studies have suggested the use of population-based AIF (pAIF) over iAIF, even though population-based arterial input function (pAIF) does not capture inter-individual physiological variation [25, 26]. pAIFs, including Tofts AIF and Parker AIF, are fitted on the average measurement of AIF samples from a group of subjects [10]. Parker AIF, a mixture of two Gaussian kernels and an exponential modulated with a sigmoid function, has similar contrast agent kinetics to the true AIF and therefore is more realistic than the Tofts AIF [10, 27]. Overall, compared with the iAIF, the pAIF is stable, has an adjustable temporal resolution, and is less affected by image artifacts and ROI bias, thereby improving the fitting effect, particularly for clinical data that inevitably contain multiple artifacts [10, 27].

The pAIF has been widely tested and successfully applied to the brain and various pelvic organs in the absence of a reliable iAIF [24–26, 28, 29]. The whole-kidney model combined with iAIF has also been successfully used for the rapid calculation of the DCE-MRI eGFR, offering comparable performance to the more complex three-compartment model compared with the gold standard measurements of eGFR, while requiring a simpler whole-kidney ROI [11, 30, 31]. Building on these studies, pAIF and the whole-kidney model, through simplified ROI drawing on the aorta and kidney, may further help to reduce the inaccuracies in iAIF and renal concentration curve measurements arising from poor image quality or suboptimal preprocessing. However, to the best of our knowledge, the feasibility of the combination of pAIF and the whole-kidney model has not been tested before.

The primary aim of this study is to streamline aortic and renal ROI delineation by combining a pAIF with a whole-kidney model, thereby minimizing inevitable artifacts and labor-intensive manual segmentation. This combination approach could simplify the DCE-MRI post-processing pipeline and reduce barriers to its clinical adoption in renal imaging. The secondary aim of the study is to compare DCE-MRI eGFR and RPF with serum eGFR (used clinically) and ASL-derived RPF, respectively.

## Materials and methods

### Patients

**Fig. 1:**
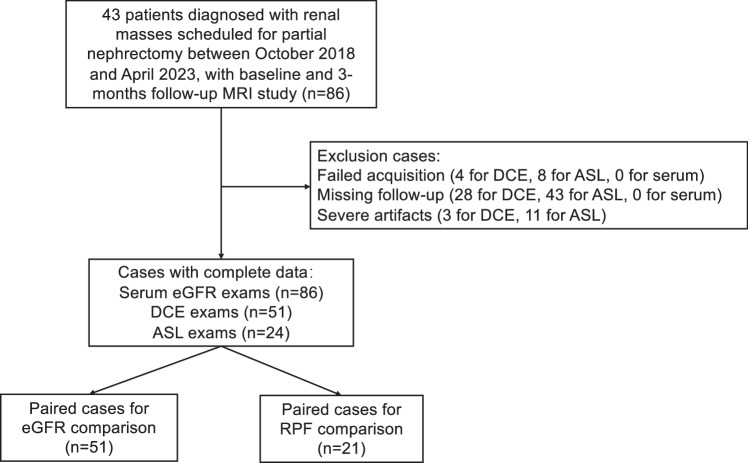
Flowchart of inclusion/exclusion of the study population. Severe artifacts (including inflow artifact and saturation artifact for DCE-MRI, registration issue caused by respiratory motion for ASL) were exclusion criteria. DCE, Dynamic contrast-enhanced; eGFR, Estimated glomerular filtration rate; MRI, Magnetic resonance imaging; RPF, Renal plasma flow

This prospective, single-center study, approved by the institutional review board, involved 43 patients diagnosed with renal masses scheduled for partial nephrectomy between October 2018 and April 2023. Informed signed consent was obtained from all participants. All patients underwent MRI before partial or radical nephrectomy, and a subset (*n* = 15) underwent follow-up MRI (including research MRI sequences) approximately three months after surgery. The chronic kidney disease (CKD) cases with serum eGFR > 30 mL/min/1.73 m^2^ underwent DCE-MRI as per the standard of care and American College of Radiology‒ACR guidelines. The characteristics of the patient population are presented in Table 1, and the flowchart of patient exclusion is shown in Fig. 1.

**Table 1:** Demographic and clinical characteristics of the study cohort

Demographic and clinical characteristics	
Age (years)	59.05 ± 11.67
Weight (kg)	87.56 ± 21.12
Height (m)	1.72 ± 0.11
BMI (kg/m^2^)	29.37 ± 5.81
Gender	
Male	30
Female	13
Race	
Caucasian	33
African-American	9
Other	1
Ethnicity	
Hispanic	4
Not Hispanic	39
CKD	
Yes (eGFR < 60 mL/min/1.73 m^2^)	13
No (eGFR ≥ 60 mL/min/1.73 m^2^)	30

The distribution of ethnicity, race, gender, and clinical characteristics, including chronic kidney disease (CKD), age, weight, height, and BMI (body mass index) for the study participants, is shown below. CKD status was determined using the serum creatinine based on the CKD-EPI formula, with values greater than 60 mL/min/1.73 m^2^ considered normal and values below 60 mL/min/1.73 m^2^ indicating CKD (*N* = 43)

### MRI protocol

All patients were imaged with a 1.5-T MRI (Aera, Siemens Healthineers, Erlangen, Germany) with an external phased-array body coil. The multiparametric MRI protocol included DCE-MRI and ASL. The DCE-MRI acquisition consisted of a free-breathing, fat-suppressed three-dimensional T1-weighted dynamic acquisition in coronal orientation (repetition time/echo time/flip angle: 4.53 ms/1.06 ms/12°, field of view 440 × 358 mm^2^, 36 slices, matrix size 192 × 125, parallel imaging factor 2 acceleration) with a temporal resolution of 5 s, and performed in three segments, interspersed with standard clinical MRI sequences. Gadobutrol (Gadavist, Bayer Healthcare US; 0.1 mmol/kg dose) and a 40 mL saline flush were intravenously injected at 2 mL/s at 25 s after the start of the DCE-MRI acquisition. ASL was also acquired in coronal orientation (repetition time/echo time: 5,000 ms/25.84 ms, field of view 280 × 140 mm^2^, 30 slices, three-dimensional gradient- and spin-echo readout), with pseudocontinuous ASL (30 label and control pairs, flip angle 28°, start inversion time = 3,000 ms, labeling duration = 1,500 ms, T1 blood = 1,250 ms), fat saturation and background suppression [32].

### DCE-MRI processing

**Fig. 2:**
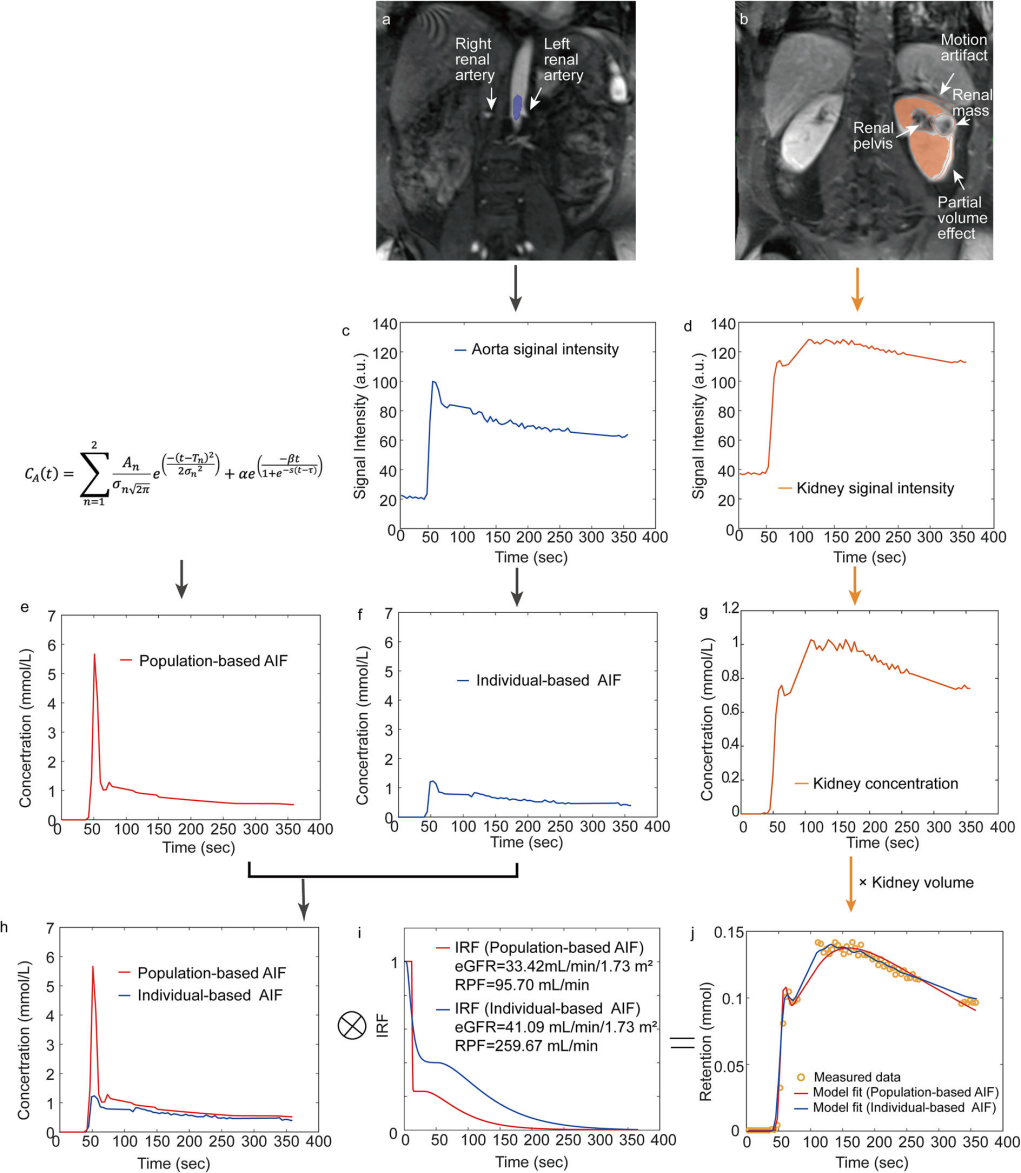
DCE-MRI processing. **a** The aorta ROI (blue) is placed slightly above the origin of the renal arteries (arrows). **b** Whole-kidney parenchyma ROI (yellow) after registration, excluding the renal mass, renal pelvis and regions affected by artifacts (arrows). **c**, **d** Signal intensity curves of the aorta (blue) and the kidney (yellow). **e** Population-based AIF generated using Parker formula (pAIF, red); **f**, **g** Individual-based AIF and renal concentration curve derived from the respective aortic and kidney signal intensity curve shown in **c** and **d**. **h** Reproduction of the AIFs shown in **e** and **f**, presented together to illustrate the markedly underestimated peak of the iAIF (blue) compared with the pAIF (red). **i** Impulse retention function (IRF) of the whole-kidney model, showing the temporal dynamics of tracer retention in the renal parenchyma. **j** The kidney retention *versus* time curve, plotting measured data (orange hollow circles) along with the fitted curves for pAIF (red) and iAIF (blue). Convolving the IRF of the kidney parenchyma with its AIFs yields the concentration-time curve in the renal parenchyma. An underestimated iAIF results in an overestimation of RPF and a seemingly reasonable eGFR, despite both retention curves fitting well. ⊗ denotes convolution

Figure 2 shows the workflow of renal DCE-MRI processing, which involves four steps: (1) ROI segmentation; (2) signal-intensity to concentration conversion; (3) individual-modified pAIF; and (4) curve fitting using the whole-kidney model. Steps 1 and 2 were performed using FireVoxel build 456 (https://firevoxel.org/), and Steps 3 and 4 were performed using MATLAB R2023a (MathWorks Inc.), as detailed below.

#### ROI segmentation

Observer 1 (a scientist with 6 years of MRI analysis experience) segmented three-dimensional ROIs and performed the registration for the aorta and every single kidney on DCE data, separately. For interobserver reliability assessment, observer 2 (a physicist with 11 years of experience) independently performed the same tasks on a subset of the cohort (*n* = 10). The aortic ROI (Fig. 2a) was placed slightly above the origin of the renal arteries. The whole-kidney parenchyma ROI (Fig. 2b) covered the entire kidney, excluding the renal pelvis, any renal masses and regions affected by artifacts. A four-dimensional autofocus coregistration (three-dimensional ROI with time dimension) was used for all DCE-MRI, with the ROI targeted to increase registration efficiency. With this step, the ROI-based signal intensity curves of the aorta and kidney can be obtained (Fig. 2c, d).

#### Signal-to-concentration conversion

In this step, the ROI-based signal intensity curves (Fig. 2c, d) were converted to concentration curves (Fig. 2f, g) by using the relationship between the longitudinal relaxation time and the concentration

1 $$C\left(t\right)=\frac{1}{{r}_{1}}\left(\frac{1}{{T}_{1}\left(t\right)}-\frac{1}{{T}_{1}\left(0\right)}\right)$$

and the spoiled gradient recalled echo signal expression, ignoring T2* effect at short echo time,

2 $${S}_{\left(t\right)}={M}_{0}\sin \alpha \frac{1-{{{e}}}^{\left(-\frac{{TR}}{{{{\rm{\top }}}}_{1}\left(t\right)}\right)}}{1-\cos \alpha {{{e}}}^{\left(-\frac{{TR}}{{{{\rm{\top }}}}_{1}\left(t\right)}\right)}}\,$$

where $C\left(t\right)$ is concentration of Gd (mmol/L), ${r}_{1}$ is longitudinal relaxivity of the contrast agent (L/mmol/s), 5.2 L/mmol/s for gadobutrol, ${T}_{1}\left(0\right)$ is precontrast longitudinal relaxation time of blood or tissue (s), with 1 s for renal parenchyma and 1.48 s for blood [33], ${T}_{1}\left(t\right)$ is postcontrast longitudinal relaxation time of blood or tissue (s), TR is the repetition time and $\alpha$ is the flip angle, M_0_ is the equilibrium magnetization, ${S}_{\left(t\right)}$ is the MRI signal intensity at each time point t.

The individual-modified population-based AIF (Fig. 2e) pAIF (Parker AIF [27]) was obtained by the Eq. (3):

3 $${C}_{A}\left(t\right)=\mathop{\sum }\limits_{n=1}^{2}\frac{{A}_{n}}{{\sigma }_{n\sqrt{2\pi }}}{{{e}}}^{\left(\frac{-{\left(t-{T}_{n}\right)}^{2}}{2{{\sigma }_{n}}^{2}}\right)}+\alpha {{{e}}}^{\left(\frac{-\beta t}{1+{{{e}}}^{-s\left(t-\tau \right)}}\right)}$$

where $\alpha$ = 1.050 mM and $\beta$ = 0.1682 min^−1^ are the amplitude and the decay constant of the exponential; *s* = 38.078 min^−1^ and $\tau$ = 0.483 min are the sigmoid width and center; *A*_1_ = 0.809 mM min, *A*_2_ = 0.330 mM min, *σ*_1_ = 0.0563 min, *σ*_2_ = 0.132 min, *T*_1_ = 0.170 min, and *T*_2_ = 0.365 min are the scales, widths, and centers of the Gaussian kernels, respectively. The time interval was set as 1 s to obtain a higher time-resolution pAIF, over the temporal resolution of 5 s of iAIF. Then, the pAIF was shifted in the time dimension to make its peak in alignment with the iAIF’s peak for each case. This adjustment was made to minimize temporal misalignment between pAIF and iAIF.

#### Model fitting

After the concentration in the aorta (iAIF, pAIF) and the kidney were obtained, the least squares fitting with the Levenberg-Marquardt algorithm was used to fit the model’s parameters, two of which are eGFR and RPF [11]. The tracer kinetic model characterized the impulse retention function R (Fig. 2i) by relating it with the kidney concentration curve *C*_*t*_ (Fig. 2g)_,_ and the aortic concentration curve *C*_*A*_ (iAIF and pAIF, Fig. 2h) by the following formula:

4 $${V}_{t}{C}_{t}(t)=\frac{{RPF}}{1-{hematocrit}}{C}_{A}(t)\otimes R(t)$$

where *V*_*t*_ is the volume of the kidney, hematocrit equals 0.4, and $\otimes$ denotes convolution [11]. Details about the whole-kidney model [11] are provided in Appendix E1. The corresponding fitted curves are presented in Fig. 2j.

#### ASL processing

As the RPF reference, the MR scanner’s built-in algorithm performs the image registration and generates an ASL-derived renal blood flow (RBF) map. Kidney ROIs for RBF were drawn by a radiologist, and ASL-derived RBF were analyzed on an averaged-ROI level. After that, RBF was converted to RPF by the formula:

5 $${RPF}={RBF}\left(1-\,{Hct}\right)$$

### Laboratory tests

As a reference, standardized serum creatinine was acquired on the day of the MR examinations. Serum eGFR was calculated by using the 2021 Chronic Kidney Disease Epidemiology Collaboration‒CKD-EPI formula [34]. All eGFR results were expressed in mL/min/1.73 m^2^.

### Statistical analysis

All statistical analyses were performed using SPSS software Version 27 (IBM SPSS). The results were shown by mean ± standard deviation (SD), and the coefficient of variation (CV, defined as the ratio of the SD to the mean) was also reported to show the variance among patients for the same method. Interobserver reproducibility was assessed by the intraclass correlation coefficient (ICC, absolute agreement, single measurement) based on a two-way mixed effects model (poor: < 0.50, moderate: 0.50–0.75, good: 0.75–0.90, excellent: > 0.90). The Mann–Whitney *U*-test was applied to compare the serum-derived eGFR values with two DCE eGFR: pAIF eGFR and iAIF eGFR. Pearson’s correlation coefficient (*r*), along with corresponding *p*-values, 95% confidence interval (CI) and linear regression, were calculated to evaluate the linear relationship between serum-derived eGFR and the DCE eGFR values obtained using pAIF and iAIF methods, respectively. Bland–Altman plots were used to assess the agreement between serum-derived eGFR and pAIF/iAIF eGFR. Bias (mean percentage difference %), SD of bias, and limits of agreement (mean ± 1.96 SD) were calculated. The above statistical analysis was also done for the comparison between ASL-derived RPF values and two DCE RPF: pAIF RPF and iAIF RPF, respectively. A significance level of 0.05 was used for all tests.

## Results

### Patients

DCE-MRI eGFR was evaluated in 51 MRI exams from 43 patients (36 baseline and 15 follow-up MRI exams), and RPF by both DCE-MRI and ASL was assessed in 21 baseline MRI exams from 21 patients (Fig. 1).

### Comparison among serum eGFR, pAIF eGFR, and iAIF eGFR

**Fig. 3:**
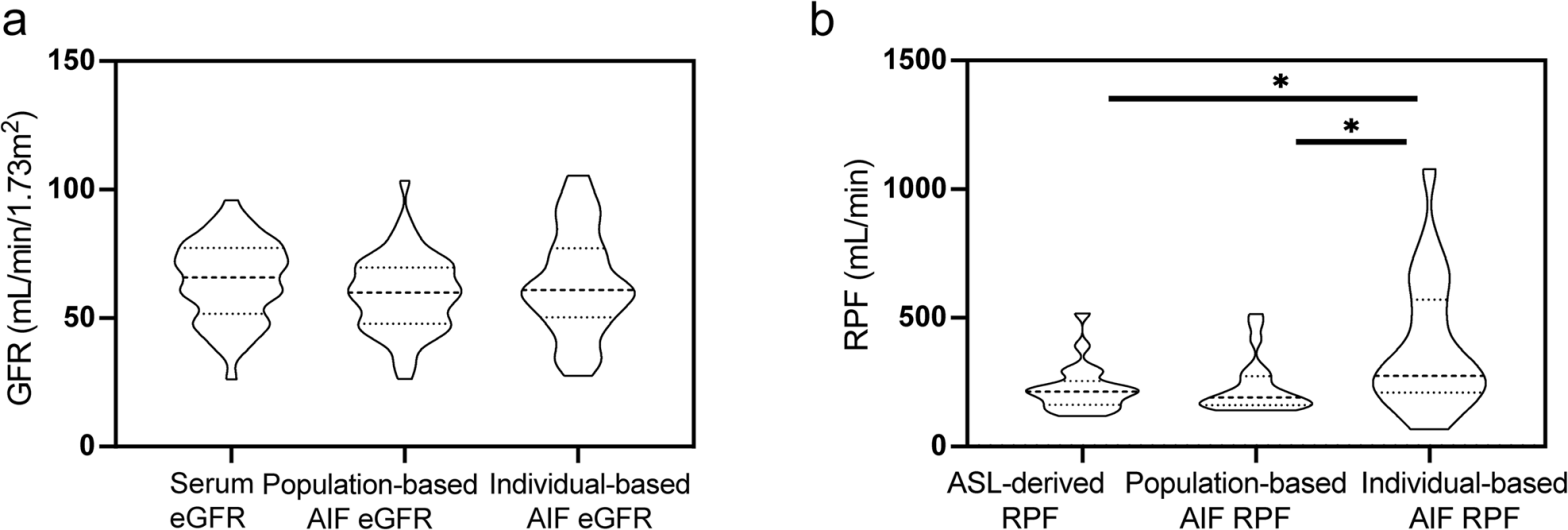
Violin plot for estimated glomerular filtration rate (eGFR) and renal plasma flow (RPF) measured by different methods. **a** The violin graph illustrates eGFR values obtained from serum creatinine, population-based AIF (pAIF), and individual-based AIF (iAIF) methods. **b** The violin graph illustrates RPF values obtained from ASL, population-based AIF (pAIF), and individual-based AIF (iAIF). The data are presented as mean ± standard deviation. Statistically significant differences between methods are indicated by asterisks (*), with *p*-values < 0.05. In violin plots, the dashed lines represent the median and quartile values, and the width of the violin shape at a certain level represents the relative occurrence frequency at that level (*n *= 51 for eGFR, *n *= 21 for RPF)

Table 2 and Fig. 3a show the comparison of eGFR values measured by three different methods: serum creatinine and clinical parameters, pAIF, and iAIF. Across the cohort of 51 exams, the mean eGFR values were 67.55 ± 17.72 mL/min/1.73 m² for the serum measurement, 59.49 ± 14.79 mL/min/1.73 m² for the pAIF measurement, and 63.60 ± 19.88 mL/min/1.73 m² for the iAIF measurement. No significant differences were found between the mean eGFR values of the three methods (*p* = 0.338 for pAIF *versus* serum eGFR, *p* = 0.705 for iAIF *versus* serum eGFR). The CVs showing interpatient heterogeneity were 0.26, 0.24, and 0.31 for serum eGFR, pAIF eGFR, and iAIF eGFR, respectively. The high CV and SD of iAIF eGFR indicated slightly higher variability with the iAIF method.

**Table 2:** Mean, standard deviation (SD), and coefficient of variation (CV) for estimated glomerular filtration rate (eGFR) and renal plasma flow (RPF) measurements based on different methods (*n* = 51 for eGFR, *n* = 21 for RPF)

	Mean	SD	CV
Serum eGFR	67.55	17.72	0.26
pAIF eGFR	59.49	14.79	0.24
iAIF eGFR	63.60	19.88	0.31
ASL-derived RPF	229.3	91.46	0.40
pAIF RPF	229.7	105.5	0.46
iAIF RPF	390.4	255.9	0.66

The units for eGFR and RPF are mL/min/1.73 m² and mL/min, respectively

*pAIF* Population-based AIF, *iAIF* Individual-based AIF

**Fig. 4:**
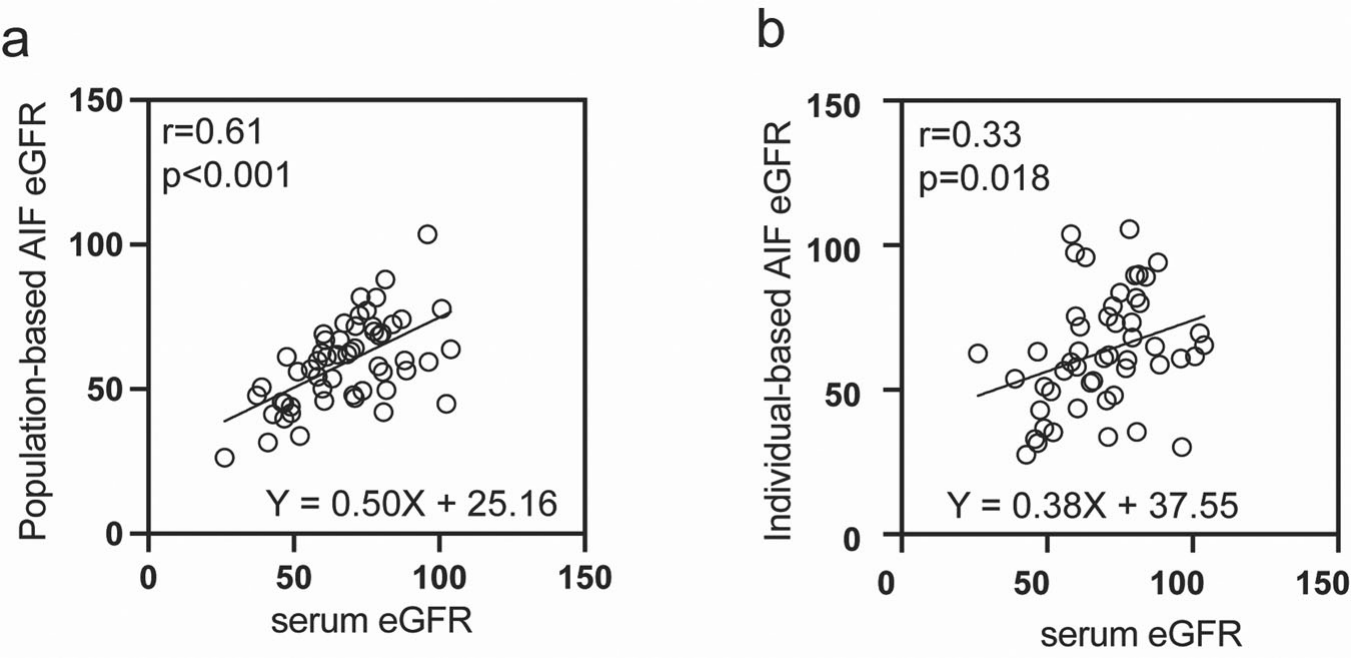
Pearson correlation plots for estimated glomerular filtration rate (eGFR) measured by different methods. **a** Correlation between serum eGFR and population-based AIF (pAIF) eGFR, showing a significant positive correlation (r = 0.61, *p* < 0.001) with the equation *Y* = 0.50*x* + 25.16. **b** Correlation between serum eGFR and individual-based AIF (iAIF) eGFR, showing a weaker correlation (*r* = 0.33, *p* = 0.018) with the equation *Y* = 0.38*x* + 37.55. (*n* = 51)

Table S1 and Fig. 4 show the correlations between serum eGFR and two DCE eGFR values. The pAIF eGFR (Fig. 4a) exhibited a moderate positive correlation with serum eGFR (*r* = 0.61, *p* < 0.001, 95% CI [0.40, 0.75]). In contrast, iAIF eGFR (Fig. 4b) had a weaker correlation with serum eGFR (*r* = 0.33, *p* = 0.018, 95% CI [0.06, 0.56]). The wider scatter of data points for iAIF eGFR suggested greater measurement variability.

**Fig. 5:**
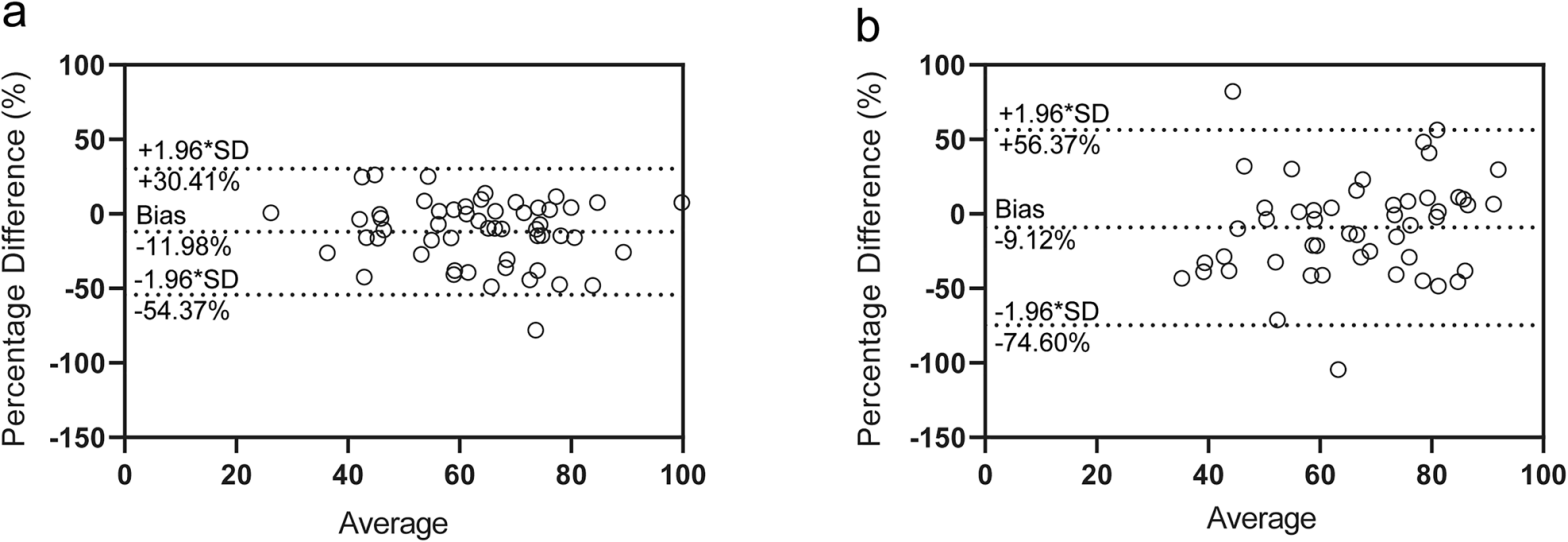
Bland-Altman agreement plots for estimated glomerular filtration rate (eGFR) measured by different methods. **a** Agreement between serum eGFR and population-based AIF (pAIF) eGFR; **b** Agreement between serum eGFR and individual-based AIF (iAIF) eGFR. The bias (mean difference %) and the limits of agreement (mean ± 1.96 * SD) are indicated by the horizontal dashed lines (*n* = 51)

Table S1 and Fig. 5 depict Bland-Altman analyses comparing serum eGFR with pAIF and iAIF eGFR, respectively. For the pAIF eGFR *versus* serum eGFR (Fig. 5a), the mean bias was -11.98%, with an SD of 21.63% and 95% limits of agreement ranging from -54.37% to 30.41%, indicating moderate variability. In contrast, the iAIF eGFR (Fig. 5b) showed a smaller mean bias (-9.12%) but a larger SD (33.41%), with 95% limits of agreement ranging from -74.60% to 56.37%. This wider range of agreement reflects increased variability with the iAIF method, consistent with findings from scatter and correlation analyses, as well as CV and SD.

### Comparison among ASL-derived RPF, pAIF RPF, and iAIF RPF

Table 2 and Fig. 3b provide the comparison of RPF values among ASL-derived RPF, pAIF RPF, and iAIF RPF. The mean RPF values for ASL-derived, pAIF, and iAIF were 229.3, 229.7, and 390.4 mL/min, respectively. The iAIF RPF was significantly higher than the ASL-derived RPF (*p* = 0.018) and the pAIF RPF (*p* = 0.012). This discrepancy was also reflected in the SD and CV, with iAIF RPF showing a much higher SD (255.9 mL/min) compared to ASL-derived (91.46 mL/min) and pAIF RPF (105.5 mL/min). Similarly, the CV for iAIF RPF was 0.66, indicating greater variability compared to ASL-derived (0.40) and pAIF (0.46) RPF values.

**Fig. 6:**
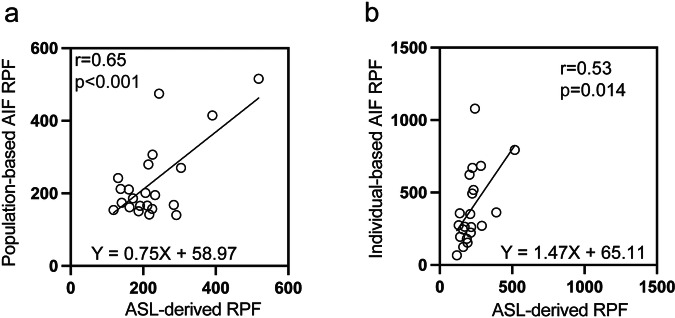
Pearson correlation plots for renal plasma flow (RPF) measured by different methods. **a** Correlation between ASL-derived RPF and population-based AIF (pAIF) RPF, showing a significant positive correlation (*r* = 0.65, *p* < 0.001) with the equation *Y* = 0.75*x* + 58.97. **b** Correlation between ASL-derived RPF and individual-based AIF (iAIF) RPF, showing a moderate positive correlation (*r* = 0.53, *p* = 0.014) with the equation *Y* = 1.47*x* + 65.11. (*n* = 21)

Table S1 and Fig. 6 illustrate the correlations between ASL-derived RPF and pAIF RPF (Fig. 6a), as well as iAIF RPF (Fig. 6b). A moderate positive correlation was observed between ASL-derived and pAIF RPF (*r* = 0.65, *p* < 0.001, 95% CI [0.32, 0.84]). In contrast, the correlation between ASL-derived and iAIF RPF was weaker (*r* = 0.53, *p* = 0.014, 95% CI [0.13, 0.78]).

**Fig. 7:**
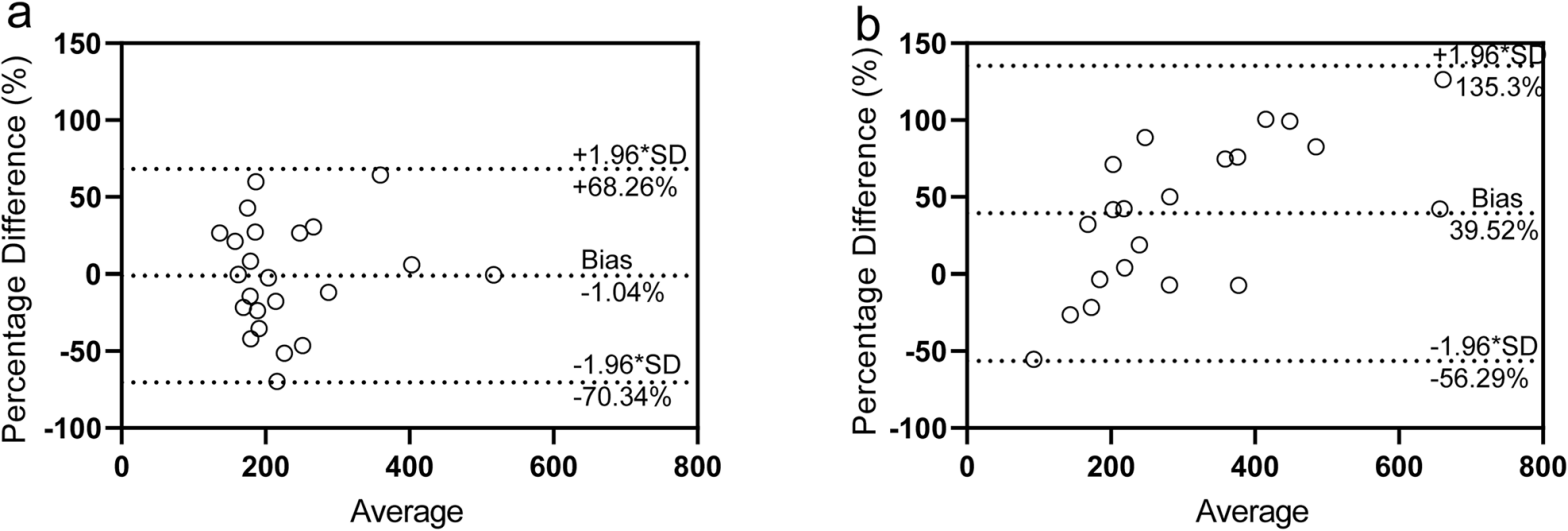
Bland-Altman agreement plots for renal plasma flow (RPF) measured by different methods. **a** Agreement between ASL-derived RPF and population-based AIF (pAIF) RPF. **b** Agreement between ASL-derived RPF and individual-based AIF (iAIF) RPF. The bias (mean difference %) and the limits of agreement (mean ± 1.96 * SD) are indicated by the horizontal dashed lines. (*n* = 21)

Table S1 and Fig. 7 present Bland-Altman analyses comparing ASL-derived RPF with pAIF RPF (Fig. 7a) and iAIF RPF (Fig. 7b). The mean bias for pAIF RPF *versus* ASL-derived RPF was -1.04%, with an SD of 35.36%, and 95% limits of agreement ranging from -70.34% to 68.25%. In contrast, the Bland-Altman plot for iAIF RPF *versus* ASL-derived RPF showed a larger mean bias of 39.52%, with an SD of 48.88% and wider 95% limits of agreement (-56.29% to 135.3%). This larger spread indicated greater variability in iAIF RPF, with a tendency to overestimate RPF, particularly at higher values.

### Interobserver reliability

Interobserver reliability between two observers for the subset of the cohort is provided in Table 3. eGFR demonstrated good interobserver agreement, with ICC values of 0.84 for the pAIF method and 0.82 for the iAIF method. RPF demonstrated excellent interobserver agreement, with ICC values of 0.95 for the pAIF method and 0.92 for the iAIF method.

**Table 3:** Intra-class correlation coefficients (ICC) for estimated glomerular filtration rate (eGFR) and renal plasma flow (RPF) from two observers

	eGFR			RPF		
	ICC	*p*	95%CI	ICC	*p*	95% CI
pAIF	0.84	< 0.001	[0.48, 0.96]	0.95	< 0.001	[0.83, 0.98]
iAIF	0.82	< 0.001	[0.23, 0.93]	0.92	< 0.001	[0.70, 0.98]

The table presents the ICCs, corresponding *p*-values, and 95% confidence interval (CI) for eGFR and RPF based on population-based AIF (pAIF) and individual-based AIF (iAIF) methods. (*n* = 10 for eGFR and RPF)

## Discussion

Accurate assessment of kidney function is essential for well-informed clinical decision-making, with GFR serving as a key indicator of overall kidney function [35]. Compared with inexpensive serum eGFR measurement and the more expensive and accurate ^99m^Tc diethylene-triamine-pentaacetate scintigraphy method, DCE-MRI offers a balanced compromise between accuracy and cost while avoiding radiation exposure in single-kidney assessment [36]. DCE-MRI can also simultaneously assess other single-kidney parameters, including RPF, filtration fraction, and mean transit time, in addition to single-kidney eGFR [3, 5, 7–9]. Considering the kidney’s innate ability to maintain GFR through hyperfiltration and compensatory hypertrophy, these additional parameters can provide a comprehensive understanding of kidney function and may provide added value for surgical planning, for potential living kidney donation, and in the characterization of unilateral native kidney disease [35, 37].

The iAIF is sensitive to ROI geometry and image artifacts, and further influences the DCE-MRI quantitative parameters [21, 23, 24]. The pAIF was designed to alleviate these limitations when a reliable iAIF is lacking. Unlike iAIF, which has a preset temporal resolution during image acquisition and thus normally requires interpolation during curve fitting, the pAIF offers the ability to modify the temporal resolution, making it more advantageous for curve fitting [38–40]. The iAIF RPF significantly overestimated RPF values, with its mean, SD, and CV nearly double those of ASL-derived and pAIF RPF. In contrast, pAIF RPF values were more consistent with ASL-derived RPF and aligned with values reported in the literature [9, 41]. Although ASL-derived RPF is not a traditional reference standard, the observed patterns still imply that the iAIF contributes to RPF overestimation. Previous studies have noted that the size, area, and location of the aorta ROI could significantly impact RPF values, but a consensus on precise aorta ROI delineation is still lacking [24]. Despite the ROI delineated by two experienced observers to avoid partial volume effect, unavoidable saturation artifact and inflow artifact in aorta ROI, together with low temporal resolution, still reduce iAIF’s peak values in some cases compared to pAIF, as shown in Fig. 2h [42]. Consequently, RPF is overestimated because its estimation is highly sensitive to the vascular phase, where iAIF peak-underestimation errors propagate directly into flow calculations [25, 26]. This phenomenon has also been reported in other organs [9, 43].

Both pAIF and iAIF methods slightly underestimated eGFR compared to serum eGFR. This aligns with the findings of Lee et al [44] and Bane et al [41], who also reported that model-derived eGFR underestimated true GFR. Considering that serum eGFR might overestimate true GFR, especially at lower GFR levels [45, 46], the whole-kidney model could slightly underestimate or accurately estimate the true GFR measured by scintigraphy methods. Since eGFR is calculated as the product of RPF and the filtration fraction at the final step, an effect of the model fit is the underestimation of impulse retention function parameters associated with the filtration fraction to compensate for the amplification of the convolution by the overestimated iAIF RPF, thereby yielding a plausible eGFR value. Such a phenomenon can be explained by the inherent uncertainty of DCE-MRI kinetic modeling, in which a combination of multiple parameters can produce almost identical fits and therefore similar solutions. [30, 47]. Even though serum eGFR is not a gold standard reference, the weaker correlation between iAIF eGFR and serum eGFR still indicates the nonnegligible impact of iAIF variability caused by artifacts [42, 48]. Overall, eGFR and RPF derived from iAIF had larger CV and SD compared to those from pAIF, as well as to their respective reference values (serum eGFR and ASL-derived RPF). Although the pAIF performs well, it should be emphasized that it does not reflect subject-specific physiological characteristics—such as variations in cardiac output or hemodynamic responses—and therefore may not be suitable for all populations. Instead, pAIF is best regarded as a reliable alternative when iAIF quality is degraded by motion, partial-volume artifacts, or uncertainties in AIF ROI placement.

The complete multi-compartment renal model divides the vascular-nephron unit into seven compartments with 17 parameters, including eGFR, RPF, and so on [13]. Common renal models are derived from this complete seven-compartment framework, with varying degrees of simplification and modification [13]. Compared to three-compartment models, which require labor-intensive cortical–medullary segmentation, and with one-compartment and other two-compartment models whose physiological assumptions are inherently limited, the whole-kidney model simplifies ROI delineation by treating the entire kidney parenchyma as a single region, while still offering a more physiologically realistic parallel two-pathway assumption [11, 15]. Previous studies have validated the reliability of the whole-kidney model by comparing it with urinary clearance measured using ^99m^Tc diethylene-triamine-pentaacetate [11]. Conlin et al compared the interobserver variability between the whole-kidney model and the three-compartment corticomedullary model, and reported slightly lower variability for whole-kidney model’s eGFR and RPF [31]. Similarly, Lee et al reported excellent interobserver ICC values for eGFR across these two models, with a range of 0.94‒0.96 [11]. Consistent with these findings, our method demonstrated good agreement for eGFR and excellent agreement for RPF, with the advantage of a more streamlined workflow. The interobserver ICC of eGFR in our study is relatively lower than that of RPF. Given that eGFR is derived from the product of RPF and filtration fraction, fitting errors in either parameter may propagate and compromise the accuracy of eGFR estimation [30].

Future developments could leverage automated whole-kidney segmentation to directly identify the kidney ROI and automatically calculate eGFR and RPF. Considering that whole-kidney segmentation avoids the need to delineate indistinct corticomedullary boundaries, particularly in diseased kidneys, this approach, combined with the maturity of automatic or semi-automatic registration and segmentation techniques, could substantially facilitate clinical translation by reducing operator dependence and minimizing variability in ROI delineation.

This study has several limitations. First, we recognize the small sample size in our study. In addition, the use of averaged T_1_ values for the kidney does not account for individual variations in tissue characteristics, potentially affecting the precision of eGFR and RPF values. Second, the clinically accepted serum eGFR and ASL-derived RPF were used as reference values, but they are not considered the true gold standard; therefore, future studies should evaluate accuracy against scintigraphy-based reference measurements, rather than relying on agreement alone. Third, although the population-based AIF is not individualized, it serves as a necessary compromise, particularly in the absence of a reliable individual-based AIF due to artifacts. Further studies in larger populations and external validation are also required.

In this study, we demonstrated the feasibility of combining a pAIF with the whole-kidney model to calculate DCE-MRI-based measures of renal function, eGFR and RPF. The proposed method demonstrates a stronger correlation and agreement for both eGFR and RPF with their respective reference values (serum eGFR and ASL-derived RPF) than the iAIF method. Also, we showed good interobserver reliability for eGFR and excellent interobserver reliability for RPF in the subset analysis. This approach enables the assessment of single-kidney eGFR and RPF through straightforward delineation of the kidney ROI. The population-based approach can reduce the inter-observer variability in AIF ROI delineation, minimize susceptibility to image artifacts, and provide AIF with higher temporal resolution. Meanwhile, the whole-kidney model can mitigate issues in corticomedullary segmentation caused by poor image quality or poor registration, further streamlining the workflow. These advantages are particularly beneficial in clinical applications.

In conclusion, our study has shown that noninvasive measurement of renal function with calculation of eGFR and RPF using DCE-MRI by combining a pAIF with a whole-kidney model is feasible with comparable measurements to other commonly used measures of renal function (serum eGFR and ASL-derived RPF). The added value of DCE-MRI measures of renal function lies in the single-kidney assessment, which is critical for surgical planning, evaluating various diseases affecting the kidney, and guiding appropriate treatment. The combination of pAIF and whole-kidney model, therefore, offers a promising and straightforward DCE-MRI postprocessing method for accessing patient-level and single-kidney function by providing key parameters such as eGFR and RPF, thereby potentially facilitating the adoption of DCE-MRI in the clinical setting.

## Supplementary information

**Additional file 1**: **Table S1**. Pearson’s correlation and Bland-Altman agreement for estimated glomerular filtration rate (eGFR) and renal plasma flow (RPF) comparison based on different methods. The table presents the Pearson correlation coefficients (r), corresponding *p*-values, and 95% confidence interval (CI), as well as bias (mean percentage difference %), standard deviation (SD) of the bias, and 95% limits of agreement (LoA) for the comparisons between different measurement pairs: population-based AIF (pAIF) eGFR vs. serum eGFR, individual-based AIF (iAIF) eGFR vs. serum eGFR, pAIF RPF vs. ASL-derived RPF, and iAIF RPF vs. ASL-derived RPF. (*n* = 51 for eGFR, *n* = 21 for RPF).

## Data Availability

The datasets analyzed during the current study are derived from clinical patient data and are therefore not publicly available due to privacy and ethical restrictions. De-identified data may be made available from the corresponding author upon reasonable request and with appropriate institutional approvals.

## Abbreviations

ASL: Arterial spin labeling
CI: Confidence interval
CKD: Chronic kidney disease
CV: Coefficient of variation
DCE-MRI: Dynamic contrast-enhanced MRI
(e)GFR: (Estimated) glomerular filtration rate
iAIF: Individual-based arterial input function
ICC: Intraclass correlation coefficient
pAIF: Population-based arterial input function
RBF: Renal blood flow
ROI: Region of interest
RPF: Renal plasma flow
SD: Standard deviation
WKPM: Whole-kidney pharmacokinetic model
