# Supplementary material for: Streamlined calculation of kidney function using dynamic contrast-enhanced MRI with population-based arterial input function and a whole-kidney model
Source: Eur Radiol Exp. 2026 Apr 21;10:49. doi: 10.1186/s41747-026-00704-3 (PMC13100090; doi:10.1186/s41747-026-00704-3)
Supplement: Supplementary file 1 — Additional file 1: Table S1. Pearson’s correlation and Bland-Altman agreement for estimated glomerular filtration rate (eGFR) and renal plasma flow (RPF) comparison based on different methods. The table presents the Pearson correlation coefficients (r), corresponding p-values, and 95% confidence interval (CI), as well as bias (mean percentage difference %), standard deviation (SD) of the bias, and 95% limits of agreement (LoA) for the comparisons between different measurement pairs: population-based AIF (pAIF) eGFR vs. serum eGFR, individual-based AIF (iAIF) eGFR vs. serum eGFR, pAIF RPF vs. ASL-derived RPF, and iAIF RPF vs. ASL-derived RPF. (n = 51 for eGFR, n = 21 for RPF). [file 41747_2026_704_MOESM1_ESM.pdf]

Streamlined calculation of renal function using DCE-MRI with population-based arterial input function and a whole-kidney model
Original article

ELECTRONIC SUPPLEMENTARY MATERIAL

Supplemental Tables

Table S1. Pearson's correlation and Bland-Altman agreement for estimated glomerular filtration rate (eGFR) and renal plasma flow (RPF) comparison based on different methods. The table presents the Pearson correlation coefficients (r), corresponding p-values, and 95% confidence interval (CI), as well as bias (mean percentage difference %), standard deviation (SD) of the bias, and 95% limits of agreement (LoA) for the comparisons between different measurement pairs: population-based AIF (pAIF) eGFR vs. serum eGFR, individual-based AIF (iAIF) eGFR vs. serum eGFR , pAIF RPF vs. ASL-derived RPF, and iAIF RPF vs. ASL-derived RPF. (n=51 for eGFR, n=21 for RPF).

	**Pearson's Correlation**			**Bland-Altman analysis**		
	**r**	**P**	**95%CI**	**Bias**	**SD of bias**	**95% LoA**
pAIF eGFR vs. serum eGFR	0.61	<0.001	[0.40,0.75]	-11.98	21.63	[-54.37,30.41]
iAIF eGFR vs. serum eGFR	0.33	0.018	[0.06,0.56]	-9.12	33.41	[-74.60,56.37]
pAIF RPF vs. ASL-derived RPF	0.65	<0.001	[0.32,0.84]	-1.04	35.36	[-70.34, 68.26]
iAIF RPF vs. ASL-derived RPF	0.53	0.014	[0.13,0.78]	39.52	48.88	[-56.29, 135.3]

Appendix E1

Whole kidney Model

This whole-kidney model involved a single impulse retention function R with two pathways to reflect the physiological state and structure of the kidney. The single R represents tracer residue of the whole-kidney parenchyma vs time curve, with the signal intensities of the cortex and medulla combined. The two pathways through which the tracer transits are as follows: some tracer molecules go through renal vascular space (the vascular pathway), and others go through renal arteries and are then filtered into tubules at the glomerulus (the tubular pathway)[11]. Thus, R of the whole kidney can be given as R(t) =1−$h_v(t)$−$h_t(t)$, where

$$h_v(t) = \mu(t - \tau_v)(1 - f)\left[1 - \left(\frac{t - \tau_v}{\tau_b} + 1\right)e^{\left(-\frac{t-\tau_v}{\tau_b}\right)}\right]$$

and

$$h_t(t) = \mu(t - \tau_t)f\left[1 - \left(\frac{t - \tau_t}{\tau_c} + 1\right)e^{\left(-\frac{t-\tau_t}{\tau_c}\right)}\right]$$

Where μ(t) is the Heaviside unit step function, and f is the filtration fraction. Parameters τ_v and τ_t are the net delay times for vascular and tubular pathways, respectively, corresponding to minimal vascular and tubular transit times, while τ_b and τ_c indicate the respective degrees of dispersion in the two pathways.

Based on the previously obtained concentration in the aorta C_A and concentration in the kidney C_t, we can derive the parameters RPF and f by the above equations. Finally, single kidney eGFR can be obtained by

$$\mathrm{eGFR} = \mathrm{f\,RPF}$$

The MRI-based eGFR and RPF were then computed as the sum of the left and right single-kidney eGFRs and RPFs.
